# Amplification of the *MYC* Gene in Osteosarcoma Secondary to Paget's Disease of Bone

**DOI:** 10.1080/13577149778209

**Published:** 1997-12

**Authors:** Takafumi Ueda, John H. Healey, Andrew G. Huvos, Marc Ladanyi

**Affiliations:** 1 Department of Surgery Orthopaedic Service Memorial Sloan-Kettering Cancer Center New York USA; 2 Department of Pathology Memorial Sloan-Kettering Cancer Center 1275 York Avenue New York NY 10021 USA

## Abstract

*Purpose*. In a previous series of 25 human osteosarcoma samples studied for *MYC* gene amplification, we found amplification in two cases (8%), including one arising in association with Paget's disease (pagetic osteosarcoma). Based on this observation, we further investigated the prevalence of *MYC* gene amplification in pagetic osteosarcomas.

*Methods. MYC* gene amplification was assessed by Southern blot analysis using frozen tissue samples in five cases of pagetic osteosarcoma and 53 cases of primary (non-pagetic) osteosarcoma. Amplification was considered present if the *MYC* copy number was six or greater.

*Results*. Three out of five patients (60%) with pagetic osteosarcoma showed *MYC* gene amplification, whereas it was present in only 5/53 patients (9.4%) with primary osteosarcoma. The incidence of *MYC* amplification in pagetic osteosarcoma was thus significantly higher than that in primary osteosarcoma (*p* = 0.016).

*Discussion*. The finding that *MYC* gene amplification may be more common in pagetic than primary osteosarcoma warrants further study and suggests pathogenetic differences between primary osteosarcomas and those arising in the setting of Paget's disease. Three of the four pagetic osteosarcomas from the present study were previously shown to be immunoreactive for p53, suggesting that p53 mutation may also be a frequent genetic lesion in these tumors.


*Sarcoma* (1997) 1, 131–134

CARFAX

ORIGINAL ARTICLE

### Amplification of the *MYC* gene in osteosarcoma secondary to Paget's disease of bone

TAKAFUMI UEDA,[1] JOHN H. HEALEY,[1] ANDREW G. HUVOS[2] & MARC LADANYI[2]

[1]*Department of Surgery, Orthopaedic Service &* [2]*Department of Pathology, Memorial Sloan-Kettering Cancer Center, New York, USA*

**Abstract**

*Purpose.* In a previous series of 25 human osteosarcoma samples studied for *MYC* gene amplification, we found amplification in two cases (8%), including one arising in association with Paget's disease (pagetic osteosarcoma). Based on this observation, we further investigated the prevalence of *MYC* gene amplification in pagetic osteosarcomas.
*Methods.* *MYC* gene amplification was assessed by Southern blot analysis using frozen tissue samples in five cases of pagetic osteosarcoma and 53 cases of primary (non-pagetic) osteosarcoma. Amplification was considered present if the *MYC* copy number was six or greater.
*Results.* Three out of five patients (60%) with pagetic osteosarcoma showed *MYC* gene amplification, whereas it was present in only 5/53 patients (9.4%) with primary osteosarcoma. The incidence of *MYC* amplification in pagetic osteosarcoma was thus significantly higher than that in primary osteosarcoma ($p = 0.016$).
*Discussion.* The finding that *MYC* gene amplification may be more common in pagetic than primary osteosarcoma warrants further study and suggests pathogenetic differences between primary osteosarcomas and those arising in the setting of Paget's disease. Three of the four pagetic osteosarcomas from the present study were previously shown to be immunoreactive for p53, suggesting that p53 mutation may also be a frequent genetic lesion in these tumors.

**Key words:** *Paget's disease of bone, osteosarcoma, amplification, p53.*

### Introduction

Paget's disease is a benign but precancerous condition affecting the mesenchymal cells of bones. Of the secondary sarcomas arising in Paget's disease of bone, osteosarcomas (pagetic osteosarcomas) are by far the most common and make up a substantial proportion of osteosarcomas of late adulthood.[1] They represent an infrequent but highly lethal complication of Paget's disease of bone. In a recent review of the Memorial Sloan-Kettering Cancer Center (MSKCC) experience with 67 cases of pagetic sarcoma, we found that sarcoma may be the initial manifestation of Paget's disease in up to 50% of patients with this complication.[2] However, remarkably little work has been performed on the molecular genetic basis of sarcomas secondary to Paget's disease.

We have recently examined the status of the *MYC* gene in a large series of osteosarcomas and found that only 2/25 patients showed *MYC* gene amplification.[3] Of these two patients, one was a 60-year-old man with Paget's disease and a tibial osteosarcoma. Based on this observation, we have further investigated the incidence of *MYC* gene amplification in pagetic osteosarcoma and compared it with that in sporadic osteosarcoma.

### Patients and methods

Five samples from five patients with pagetic osteosarcoma were identified in our osteosarcoma frozen tissue bank. We also examined 58 frozen tissue samples from 53 patients with primary (non-pagetic) osteosarcoma as a control group. All these cases were confirmed as osteosarcoma by histological review (AGH). The results of 25 patients (24 non-pagetic, one pagetic osteosarcoma) included in the current series have been reported previously.[3] All five samples of pagetic osteosarcoma were obtained from primary tumors in patients with previously diagnosed Paget's disease of bone, while the sporadic (non-pagetic) osteosarcoma samples were obtained from 41 primary and 17 metastatic tumors.

Correspondence to: M. Ladanyi, Department of Pathology, Memorial Sloan-Kettering Cancer Center, 1275 York Avenue, New York, NY 10021, USA. Tel: + 212 639 6369; Fax: + 212 717 3515; E-mail: ladanyim@mskcc.org.

1357-714X/97/030131–04 $9.00 © 1997 Carfax Publishing Ltd

There were three male and two female patients with an age range of 60–81 years (mean 71.2 years) among the pagetic osteosarcomas, and 33 male and 20 female patients with an age range of 5–83 years (mean 25.4 years) in non-pagetic osteosarcomas. The primary sites of the tumors included the tibia (two cases), femur (one), humerus (one) and ilium (one) in the pagetic osteosarcomas, and the femur (18), tibia (13), humerus (four), ilium (four) and other sites (14) in the non-pagetic osteosarcomas.

*MYC* gene amplification was assessed essentially as previously described.[3] In brief, we performed quantitative comparison of the Southern blot hybridization signal of a genomic *MYC* exon-1 probe (Xho I–Xba I fragment) to that of a probe for a reference gene on chromosome 8, neurofilament light polypeptide (NEFL, band 8p21). Signals were quantified directly on a BioRad Phosphorimager (Hercules, CA, USA). A reference probe from the same chromosome was used to control for the non-specific effect of polyploidization on the quantitation of gene copy number.[4] HL-60 cell line DNA and human placental tissue DNA were used as positive and negative controls for *MYC* gene amplification, respectively. The *MYC* gene is amplified approximately 16-fold in the HL-60 cell line.[5] To score a sample as positive, we used a cut-off point of six copies per cell for the *MYC* gene amplification normalized to the signal in human placenta. Fractional amplification values were rounded off to the nearest whole number.

### Results

*MYC* gene amplification was detected in 3/5 patients (60%) with pagetic osteosarcoma (Fig. 1). A quantitative analysis of pagetic osteosarcoma cases 1, 2 and 3 showed amplification levels of 8, 11 and 6 copies per cell, respectively. The other two pagetic osteosarcoma cases, 4 and 5, showed no amplification. In contrast, only 5/53 patients (9.4%) with sporadic (non-pagetic) osteosarcoma revealed *MYC* gene amplification, ranging from 6 to 15 copies (mean 10 copies). The incidence of amplification in pagetic osteosarcoma was significantly higher than that in primary (non-pagetic) osteosarcoma (60% vs 9.4%, $p = 0.016$ odds ratio: 14.4) by two-tailed Fisher's exact test (Table 1).

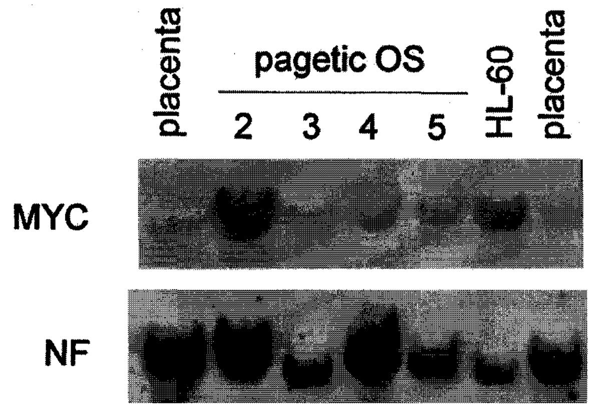

**Fig. 1.** *MYC gene amplification analysis by Southern blotting. Quantitative comparison of the hybridization signal for* MYC *and the reference probe NF (neurofilament) showed 11 and 6 copies per cell of the* MYC *gene in pagetic osteosarcoma cases 2, and 3, respectively. Amplification is visually apparent in case 2, but required quantitative confirmation in case 3. Quantitative analysis in case 5 showed five copies of* MYC *and it was scored as negative for amplification. Pagetic osteosarcoma cases 4 and 5 showed no amplification. HL-60 and human placenta, respectively, were used as positive and negative controls for amplification.*

In previous studies, including some of these cases, we found that 3/4 pagetic osteosarcomas showed p53 over-expression, but none showed amplification of *MDM2* (Table 1).[6]

Clinical features such as sex and primary site were

**Table 1.** *Data on sporadic osteosarcomas with* MYC *amplification and osteosarcomas arising in Paget's disease*

| Case number | Age/sex | Primary site | Histology | Sample type | p53 IHC | MYC* |
|---|---|---|---|---|---|---|
| 1. Sporadic: | | | | | | |
| OS6 | 7/F | Femur | Osteoblastic | P | – | 7.0 |
| OS15 | 37/M | Femur | Fibrohistiocytic | P | – | 3.0 |
| OS39 | 17/F | Tibia | Osteoblastic | P | + | 7.5 |
| OS87 | 33/F | Ulna | Osteoblastic | P | ND | 3.0 |
| OS108 | 8/M | Tibia | Chondroblastic | P | ND | 4.5 |
| 2. Pagetic: | | | | | | |
| case 1** | 60/M | Tibia | Osteoblastic | P | – | 4.0 |
| case 2 | 72/M | Femur | Giant-cell rich | P | + | 5.5 |
| case 3 | 76/M | Humerus | Fibrohistiocytic | LR | + | 3.0 |
| case 4 | 81/F | Tibia | Fibrohistiocytic | P | ND | 1.0 |
| case 5 | 67/F | Ilium | Osteoblastic | P | + | 1.0 |

*Fold amplification.
**Tumor OS22 in ref. 3.
P: primary; LR: local recurrence; p53 IHC: immunohistochemistry for p53 accumulation using antibodies D07 and 1801; ND: not done.

not significantly different between pagetic and non-pagetic osteosarcoma patients, but, as expected, the former were older (71.2 years in pagetic vs 25.4 years in non-pagetic osteosarcoma). Among the sporadic osteosarcoma patients, there was also no significant difference in age distribution between cases with and without *MYC* gene amplification. Case 4 also had a history of Paget's disease of the nipple due to extensive underlying intraductal carcinoma, representing a rare coincidence of these two eponymous conditions.

### Discussion

The *MYC* proto-oncogene, which maps to chromosome band 8q24, encodes a transcription factor containing dimerization motifs (helix–loop–helix (HLH) and leucine zipper (LZ)).[7] Normally, MYC dimerizes with another HLH/LZ protein, MAX, and interacts with promoters containing the sequence CACGTG. The spectrum of genes whose transcription is physiologically regulated by MYC is a subject of active study. At the cellular level, MYC is known to drive cell proliferation in response to extracellular signals and to promote apoptosis in proliferating cells upon withdrawal of the same signals.[7] Recently, the cell cycle phosphatase CDC25 has been identified as a direct target of transcriptional activation by MYC, providing the first clear link between MYC deregulation and cell cycle activation.[8]

The present study of frozen tumor samples of pagetic and non-pagetic osteosarcomas suggests that *MYC* gene amplification is a common molecular genetic alteration in pagetic osteosarcoma compared to its low incidence in sporadic osteosarcoma. To our knowledge, this is the first study of molecular genetic alterations in pagetic osteosarcomas.

That the *MYC* proto-oncogene (formerly *c-myc*) plays an important role in osteosarcoma has been noted in various animal models and osteosarcoma cell lines, usually in the form of amplification.[9–12] In the murine SEWA osteosarcoma cell line, tumorigenicity is proportional to the degree of *MYC* gene amplification.[13] However, few samples of human osteosarcoma have been studied for *MYC* gene amplification. Masuda *et al.*[14] found *MYC* amplification in 1/3 osteosarcomas, and Ikeda *et al.*[15] in two of four high-grade pediatric osteosarcomas. In an earlier series, we found *MYC* gene amplification in only 2/25 osteosarcomas (8%), one of which was a pagetic osteosarcoma.[3] More recently, another group found *MYC* amplification in 1/8 osteosarcomas (13%) of unspecified type.[16] In this study, we found *MYC* amplification in 9.4% of sporadic osteosarcomas. These data suggest that the true prevalence of *MYC* gene amplification in sporadic osteosarcoma is lower than expected from limited earlier studies. This discrepancy may be due to the method of quantification and to the use of surgical specimens, where amplification may be harder to detect because of admixed non-tumor tissue. Nevertheless, in the present series, pagetic osteosarcomas revealed a higher prevalence of *MYC* gene amplification (60%), suggesting a more important role for *MYC* gene amplification in pagetic osteosarcomas than in primary (non-pagetic) osteosarcomas.

In addition to the potential biological significance of *MYC* gene amplification, its presence may also be relevant clinically. In some tumor types, the presence of amplification of specific genes predicts a poorer prognosis.[17,18] Evidence is now accumulating that, likewise, *MYC* gene amplification is associated with poorer prognosis in musculoskeletal sarcomas including osteosarcomas.[16,19] Correspondingly, in some human osteosarcoma cell lines, *MYC* gene amplification is associated with higher growth rate.[20,21] Finally, in two cases of pagetic osteosarcoma, we found a coincidence of *MYC* amplification and p53 over-expression (indicative of p53 point mutation), in contrast to a previous study of these two alterations in sarcomas.[22]

### Acknowledgement

Takafumi Ueda was a visiting research fellow from the Department of Orthopaedic Surgery, Osaka Medical Center for Cancer and Cardiovascular Diseases, Osaka, Japan.

### References

1 Huvos AG. *Bone tumors—diagnosis, treatment, and prognosis.* 2nd edn. Philadelphia: WB Saunders, 1991:201–22.
2 Healey JH, Buss D. Radiation and pagetic osteogenic sarcomas. *Clin Orthop* 1991; 270:128–34.
3 Ladanyi M, Park CK, Lewis R, *et al.* Sporadic amplification of the *MYC* gene in human osteosarcomas. *Diag Mol Pathol* 1993; 2:163–7.
4 Schwab M, Amler LC. Amplification of cellular oncogenes: a predictor of clinical outcome in human cancer. *Genes Chromos Cancer* 1990; 1:181–93.
5 Dalla-Favera R, Wong-Staal F, Gallo RC. *onc* gene amplification in promyelocytic leukemia cell line HL-60 and primary leukaemic cells of the same patient. *Nature* 1982; 299:61–3.
6 Lonardo F, Ueda T, Huvos AG, *et al.* p53 and MDM2 alterations in osteosarcomas. Correlation with clinicopathologic features and proliferative rate. *Cancer* 1997; 79:1541–7.
7 Koskinen PJ, Alitalo K. Role of *myc* amplification and overexpression in cell growth, differentiation and death. *Semin Cancer Biol* 1993; 4:3–12.
8 Galaktionov K, Chen X, Beach D. Cdc25 cell-cycle phosphatase as a target of *c-myc*. *Nature* 1996; 382:511–17.
9 Isfort RJ, Cody DB, Lovell G, *et al.* Comparable oncogene and tumor suppressor gene alterations in rat and human osteosarcomas. *Prog Clin Biol Res* 1992; 376:321–30.

10 Kochevar DT, Kochevar J, Garrett L. Low level amplification of *c-sis* and *c-myc* in a spontaneous osteosarcoma model. *Cancer Lett* 1990; 53:213–22.

11 Schwab M, Ramsay G, Alitalo K, *et al.* Amplification and enhanced expression of the *c-myc* oncogene in mouse SEWA tumor cells. *Nature* 1985; 315:345–7.

12 Sturm SA, Strauss PG, Adolph S, *et al.* Amplification and rearrangement of *c-myc* in radiation-induced murine osteosarcomas. *Cancer Res* 1990; 50:4146–53.

13 Martinsson T, Stahl F, Pollwein P, *et al.* Tumorigenicity of SEWA murine cells correlates with degree of *c-myc* amplification. *Oncogene* 1988; 3:437–41.

14 Masuda H, Battifora H, Yokota J, *et al.* Specificity of proto-oncogene amplification in human malignant diseases. *Mol Biol Med* 1987; 4:213–27.

15 Lkeda S, Sumii H, Akiyama K, *et al.* Amplification of both *c-myc* and *c-raf-1* oncogenes in a human osteosarcoma. *Jpn J Cancer Res* 1989; 80:6–9.

16 Barrios C, Castresana JS, Kreicbergs A. Clinicopathologic correlations and short-term prognosis in musculoskeletal sarcoma with *c-myc* oncogene amplification. *Am J Clin Oncol* 1994; 17:273–6.

17 Perren TJ. *C-erbB-2* oncogene as a prognostic marker in breast cancer. *Br J Cancer* 1991; 63:328–32.

18 Seeger RC, Brodeur GM, Sather H, *et al.* Association of multiple copies of the *N-myc* oncogene with rapid progression of neuroblastomas. *N Engl J Med* 1985; 313:1111–16.

19 Ozaki T, Ikeda S, Kawai A, *et al.* Alterations of retinoblastoma susceptibility gene accompanied by *c-myc* amplification in human bone and soft tissue tumors. *Cell Mol Biol* 1993; 39:235–42.

20 Bogenmann E, Moghadam H, DeClerck YA, *et al.* *C-myc* amplification and expression in newly established human osteosarcoma cell lines. *Cancer Res* 1987; 47:3808–14.

21 Kawai A, Ozaki T, Ikeda S, *et al.* Two distinct cell lines derived from a human osteosarcoma. *J Cancer Res Clin Oncol* 1989; 115:531–6.

22 Castresana JS, Barrios C, Gomez L, *et al.* No association between *c-myc* amplification and *TP53* mutation in sarcoma tumorigenesis. *Cancer Genet Cytogenet* 1994; 76:47–9.
